# Ethnopharmacological studies of indigenous plants in Kel village, Neelum Valley, Azad Kashmir, Pakistan

**DOI:** 10.1186/s13002-017-0196-1

**Published:** 2017-12-01

**Authors:** Khawaja Shafique Ahmad, Abdul Hamid, Fahim Nawaz, Mansoor Hameed, Farooq Ahmad, Jiabin Deng, Noreen Akhtar, Ambreen Wazarat, Sehrish Mahroof

**Affiliations:** 1 Department of Botany, University of Poonch Rawalakot, Azad Kashmir, 12350 Pakistan; 2 Department of Horticulture, University of Poonch Rawalakot, Azad Kashmir, 12350 Pakistan; 3 Department of Agronomy, Muhammad Nawaz Sharif, University of Agriculture, Multan, Pakistan; 4 Department of Botany, University of Agriculture, Faisalabad, Pakistan; 5 School of Geography and Tourism, Guizhou Education University, Guiyang, China; 6 Department of Botany, G.C. Women University, Faisalabad, 38000 Pakistan

**Keywords:** Ethnobotany, Use value, Informant consensus factor, Jaccard index, Neelum Valley, Western Himalaya

## Abstract

**Background:**

This explorative study was undertaken for the first time in Kel village located in the Upper Neelum Valley, Azad Kashmir, Pakistan. The purpose was to document the indigenous knowledge of the native people used in the preparation of herbal medicines.

**Methods:**

To get the data on traditional uses of medicinal plants, 20 informants were interviewed. Quantitative ethnobotanical indices, i.e., use value (UV), relative frequencies of citation (RFC), informant consensus factor (Fic), fidelity level (FL), data matrix ranking (DMR), preference ranking (PR), and jaccard index (JI), were calculated for the recorded medicinal plants.

**Results:**

A total of 50 medicinal plants belonging to 33 families used in 13 disease categories were documented. Leaves were the frequently used plant parts, and decoction was the commonly used method for herbal medicine. Plants with high use value were *Berberis lycium* (2.05), *Impatiens glandulifera* (1.95), *Artemisia scoparia* (1.75), *Ageratum conozoides* (1.75), and *Achillea millefolium* (1.7). The highest RFC value was calculated for *Berberis lycium* (0.75), *Cynoglossum lanceolatum* (0.65), and *Impatiens glandulifera* and *Achillea millefolium* (0.60 each). The maximum informant consensus factor was for urinary system, cardiac diseases, baldness, and abortion and miscarriage (1.00). *Berberis lyceum* (95%) used in jaundice, hepatitis, typhoid, fever, and tuberculosis disorders. Plants with maximum fidelity level (FL) were *Berberis lycium* (95%) followed by *Dioscorea bulbifera*, *Impatiens glandulifera*, and *Artemisia vulgaris* (90%). *Olea ferruginea* was the most multipurpose plant and exports (21.2%) was the leading threat in the area. The pearson correlation coefficient (0.500) showed a positive correlation between the use value and relative frequency of citation.

**Conclusion:**

The present study provides useful information about traditional uses of medicinal plants used by local communities in different ailments. The plants with the highest use values could be employed in pharmacological research and biotechnological approaches in order to achieve adequate revenue. Some of the plants in the study area are facing high threats of becoming rare, and conservation initiatives are needed to conserve them for sustainable management in the region.

## Background

Since the origin of human civilization, medicinal plants are considered important natural resources [1]. Utilization of local plants by traditional healers has always been a low-priced and reachable source of the poor communities [2]. The indigenous knowledge of traditional drugs based on the use of plants by the local communities has been involved in scientific discipline for centuries and travels through generations from older to younger ones [3]. Rapid urbanization and dependence of mankind on modern health care systems has resulted in a decline in the traditional knowledge on the one hand, but on the other hand, utilization of plants in modern medicine has significantly increased, but this folk system still prevails in the rural communities [4].

Investigations on the ethnomedicinal uses of plants by the indigenous people are often noteworthy; as a result, they provide a gateway for the study of the new drugs source from the herbal origin [2, 4, 5]. The usage of medicinal plants to battle with various ailments is as old as human civilization [6]. Plant services to mankind are not limited to food, clothing, and shelter but their use of health care, decoration, and spiritual ceremonies is also well known [7]. About 20% of the entire plants found in this world are utilized for medicinal purposes to treat ailments in human beings [8].

The study of medicinal plants through a qualitative survey method has a very old history but the interest in numerical ethnobotany has established progressively in the last couple of decades [9]. For this, earlier authors have developed various indices that measure cultural and medicinal importance of plants quantitatively [10, 11]. These indices were utilized to measure uses of plants for different purposes such as food, veterinary medicine and, particularly, to cure human diseases [12, 13]. More or less, one common purpose of these quantitative ethnobotanical indices was to determine the importance of plants for ethnic and indigenous people [9].

More than 75% of Pakistani people depend on traditional medicines for all or most of its medicinal needs [14], and about 600 plant species are being used medicinally or traditionally in Pakistan [15]. However, the majority of the medicinal plants are confined to the north and west regions of Pakistan due to the presence of the Himalaya, which is considered a hub for a wide range of medicinal plants [16], harboring about 8000 species of flowering plants [17].

Ethnopharmacological-based studies conducted in the upper parts of the Neelum Valley are missing. This may be because of the topographical challenges of the area that comprises of high mountains that limit the access of researchers to explore the area. Kel village lies in the upper parts of the Neelum Valley in the western Himalaya. The area has a rich wealth in medicinal plants because of its conductive environment but this area remained unexplored due to its remoteness, difficult geographic conditions, and poor access through roads. This is the first effort in this region to provide quantitative ethnobotanical data employed by indigenous people. The present study was commenced with the objectives of (i) enlisting native medicinal flora and (ii) recording the aboriginal medicinal information of these flora along with their mode of preparation; additionally, we also commenced different measureable tools (iii) to find out the correlation between plants and ethnomedicinal use and (iv) to provide baseline data for forthcoming phytochemical and pharmacological research by the application of quantitative indices.

## Methods

### Study area

Neelum valley is located in the north-east part of Muzaffarabad at 900–6325 m elevation above sea level. It lies between 73°–75° E longitude and 32°–35° N latitude, covering an area of 3737 km^2^ [4]. The Kel is a lush green village, has a hill station, and a tourist spot in the upper part of the Neelum Valley, and lies between 34.8063° N 74.3460° E at an altitude of 2554 m. The climate of the study area is of temperate type where the winter is very cold (average temperature − 2.0 °C) and the summer is pleasant (average temperature 37.0 °C), and the average rainfall is 165 cm annually [18].

The region is characterized by its remoteness, long distance from urban centers, difficult mountainous terrain, and a lack of government services, including modern health care facilities. The area has poorly developed road and other infrastructure. The people of the area rely on sustainable agriculture. Main crops include corn (*Zea mays* L.), turnip (*Brasica rapa* L.), and bean (*Phaseolus vulgaris* L.) in an integrated system. A high proportion of local people are associated with livestock. A number of the main occupations are associated with summer tourism, including rest house managers, tour guides, shop keepers, restaurant workers, and jeep drivers. In light of these demographic changes, it is vital to document the local knowledge of medicinal plant usage in this area before such information declines or is lost completely.

### Data collection

Ethnobotanical fieldwork of this quantitative study was conducted during April 2016 to October 2016 following the method of [19]. Standard ethnobotanical methods such as participant observation and open and semi-structured interviews were used to gather the information [20, 21]. All the participants were native of the study area. The purpose, method, and nature of the research were explained before the informants and prior informed consent (PIC) were strictly followed during the field survey. A sum of 20 key informants, comprising of 14 men and 6 women, were selected after the initial survey and many discussions. Demographic information is presented in Table 1 which showed that 20 key informants were selected for the interviews, and out of these, four were between the ages of 30–45, six were ranging 46–60, eight were between the age of 61–75, and two were above 76. Educational status of the informant revealed that there were illiterate (40%), primary and middle (20% each), and secondary and graduate (10% each). Occupation wise, there were housewives (20%), shopkeepers and farmers (15%), labors and teachers (10% each), and local healers (30%). Selection of the informants was made based on their popularity in the study area. They were well known in the area due to their expertise to perform as a medicinal practitioner and had sound information on medicinal plants. In addition to this, information obtained from female informants about the use of indigenous plant in different ailments was compared with the information obtained from the male informants. It was observed that female informants have more knowledge about the utilization of local plants in the preparation and administration of local drugs, which mirror their part in household administration and infection treatment with a specific end goal to keep the family healthy. Meanwhile, their role as a plant collector particularly in rough and steep mountainous tracts of the area was found to be less as compared to men and traditional healers. The same findings were made during the ethnobotanical survey by [22] in the high mountainous region of Chail valley, District Swat, Pakistan, and [23] in Batan Island, Philippines, who additionally found that the ladies assume an imperative part in the arrangement of customary medications utilizing restorative plants. Information was gathered from the informants by using the standard method of [24].

**Table 1:** Demographic information of the respondents

Variables	Demographic categories	Total	Percentages (%)
Gender	Men	14	70
	Women	6	30
Age groups	30–45	4	20
	46–60	6	30
	61–75	8	40
	76 and above	2	10
Educational attainment	Illiterate	8	40
	Primary	4	20
	Middle	4	20
	Secondary	2	10
	graduate	2	10
Occupation	Housewives	4	20
	Shopkeepers	3	15
	Farmers	3	15
	Labors	2	10
	Primary teachers	2	10
	Local healers	6	30

Ethical approval for this study was obtained from the headmen of the studied areas. All respondents were asked to sign a Prior Informed Consent (PIC) form after the objectives and possible consequences of the study had been explained. The PIC form was translated into the Urdu language; however, participants were not subjected to any clinical treatment.

### Data preservation

Plant specimens for each species were collected, pressed, dried. Before mounted on the herbarium sheets they were sprayed with the help of a preservative 1% HgCl_2_ solution. For identification of the plant specimens, authentication of data, botanical names and families of each plant specimen were confirmed with the help of herbaria comparison, taxonomic literature, manuals and Flora of Pakistan [25]. Whereas the International Plant Name Index (IPNI), Scopus, Web of Science and Google Scholar, Catalog of vascular plants of West Pakistan and Kashmir [26] were also consulted to obtain correct botanical names. Jain and Rao [27] was followed to assign voucher specimens number to each plant and mounting of plants on herbarium sheet was done by standard herbarium techniques [28]. Plants were then deposited at the Herbarium Department of Botany, University of Poonch Rawalakot with voucher numbers.

### Quantitative ethnobotanical data analysis

Ethnobotanical data was collected using various quantitative indices including use value (UV), relative frequency of citation (RFC), informant consensus factor (Fic), fidelity level (FL), data matrix ranking (DMR), priority ranking (PR), and Jaccard index (JI). Association between indices was tested using correlation analysis.

### UVs

The use value is a quantitative measure of the relative importance of the species. It is used to record the most important plant species in the study area based upon the number of uses cited by the number of people. It was calculated following the standard protocol of [29].

$$ \mathrm{UV}=\genfrac{}{}{0pt}{}{\left(\Sigma \mathrm{Ui}\right)}{N} $$

where Ui is the number of use reports cited by each informant for a given species and *N* is the total number of informants.

### RFC

The relative frequency of citation was calculated to assess the incidence of one particular plant species used for the treatment of particular disease or disease category. It was calculated by using following formula as described by [30].

$$ \mathrm{RFC}=\frac{\mathrm{FC}}{N} $$

where FC is the number of informants reporting particular uses of a species and *N* is the total number of informants.

### Fic

Informant consensus factor was used to check the similarity on the informant’s information for each use category and also to check the authenticity of the work by using the following formula:

$$ \mathrm{Fic}=\frac{N_{ur}-{N}_t}{N_{ur}-1} $$

where *N*_ur_ is the number of use reports in each category, *N*_t_ is the number of species used. The factor provides a range of 0 to 1 [31].

### FL

The fidelity level (FL) was determined for the most frequently used category by calculating the percentage of informants claiming the use of a certain plant for the same major purpose [28].

$$ \mathrm{FL}=\frac{\mathrm{Np}}{N}\times 100 $$

where Np is the number of informants that claim a use of a plant species for a particular use and *N* is the number of informants that use the plants.

### DMR

Data matrix ranking was conducted using eight multi-use plants commonly reported by informants following protocol as described by [32]. Preferentially selected 10 informants were asked to give value to each trait on the basis of benefits obtained from each plant.

### PR

Threats to plants of the study area were assessed by obtaining the data from six selected key informants following [20, 28]. This information is beneficial to determine the leading threats to native flora and also helps to suggest the necessary appropriate conservation measures.

### JI

To find out the resemblance of indigenous data among various ethnic groups, this study was compared with already published work from the surrounding areas by using the Jaccard index [33].

$$ \mathrm{JI}=\frac{c\times 100}{\left(a+b\right)-c} $$

In this equation, *a* is the number of species of our area, *b* is the number of species of the neighboring area, and *c* is the number of common species in both areas.

### Pearson correlation

To find the correlation between RFC and UV values, Pearson correlation was carried out using SPSS version 16.00 Statistical software. The *R* ^2^ was determined to assess species variation in terms of used value.

## Results and discussion

### Documentation of medicinal plants

**Fig. 1:**
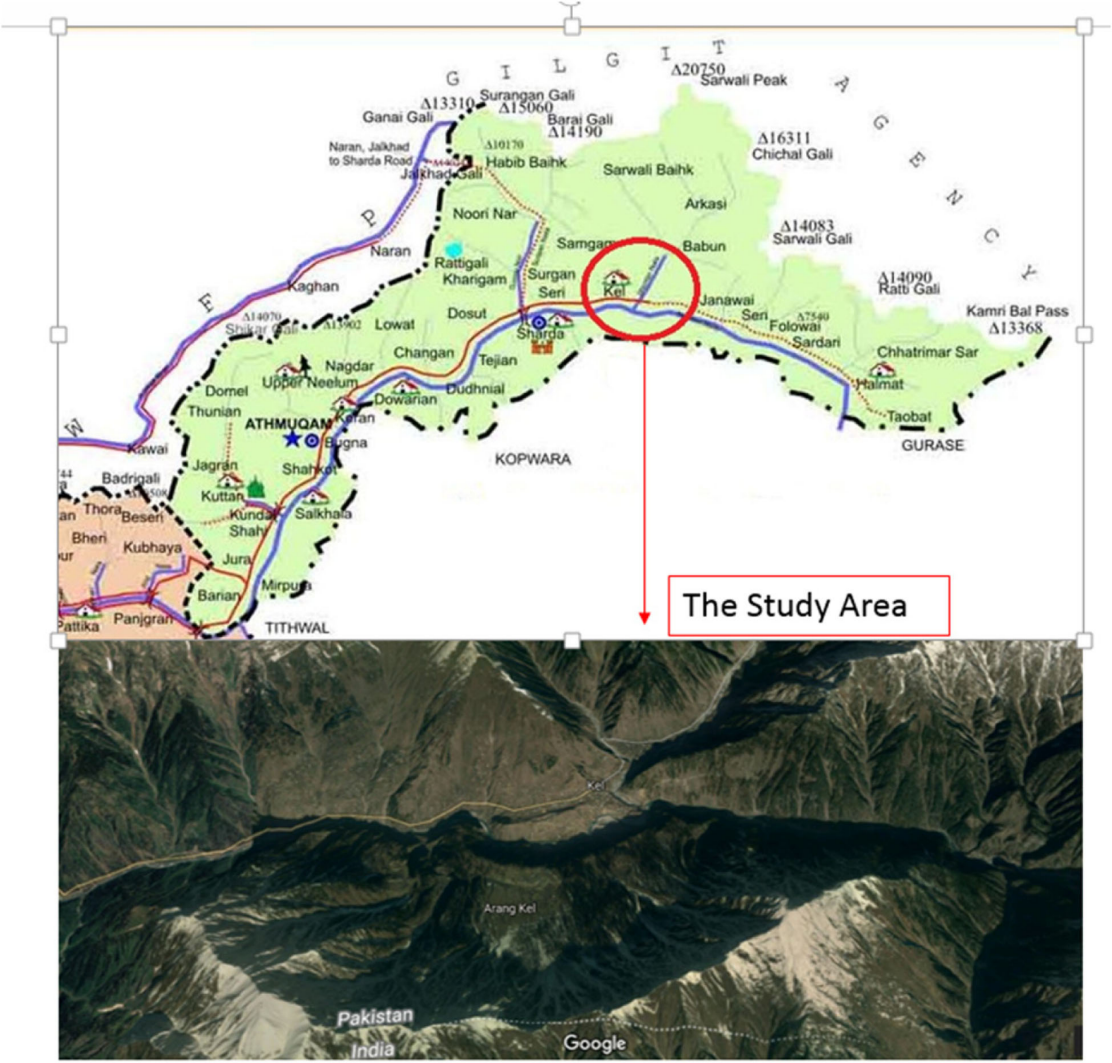
Map of the studied area with locations within the upper part of District Neelum

The present study was conducted in Kel village and its allied areas in the upper parts of Neelum Valley, which is far and remote area of Azad Jammu and Kashmir (Fig. 1). Each species recorded in this study was used in curing different diseases by the local people of the area. Table 1 presents the demographic data of informants while Table 2 provided detailed information of 50 plant species belonging to 33 families used against 13 disease categories. The present work is an effort to enumerate the ethnobotanical data based on which extensively used, high-rated medicinal plant can be selected for searching bioactive compounds to treat ailments. There are only few studies from the area of Kashmir and adjoining areas [1, 2, 18, 34–37]. A total of 50 species were documented to manage miscellaneous human and livestock ailments in the study area. The exercise to utilize the medicinal plants for the welfare of the native people is being used by many rural communities in Pakistan [21, 38].

**Table 2:** Medicinal plants used in Kel, Neelum Valley, Azad Kashmir, Pakistan

Family	Plant species/voucher No.	Part(s) used	∑Ui	UVs	FC	RFC	Ailments	Mode of administration
Apiaeceae	*Anethum graveolens* L. (dill)/KNV-221	Fruit	16	0.8	8	0.4	Digestive disorders	Extract, powder
Araliaceae	*Hedera nepalensis* K.Koch/KNV − 219	Leaves	22	1.1	9	0.45	Ulcer, dyspepsia, skin allergies	Decoction, juice
Asparagaceae	*Asparagus adsendens* Roxb./RT-220	Root	25	1.25	10	0.5	Dysentery, diarrhea	Powder
Asteraceae	*Achillea millefolium* L./KNV-242	Leaves, fruit	34	1.7	12	0.6	Wound healing, digestion, earache, toothache, tuberculosis	Infusion, juice, eat
	*Ageratum conozoides* L./KNV-243	Whole plant	35	1.75	11	0.55	Leprosy, bone dislocation, fever	Infusion, juice, decoction
	*Artemisia scoparia* Waldst& Kit./KNV-244	Leaves	35	1.75	8	0.4	Hepatitis, jaundice, stomach disorders	Infusion
	*Artemisia vulgaris* L./KNV-245	Leaves and root	33	1.65	8	0.4	Liver pain, stomach disorder	Juice, extract
	*Inula grandiflora* Willd./KNV-246	Whole plant	27	1.35	5	0.25	Cancer, skin allergies	Extract
	*Jurinea dolomiaea* Boiss./KNV-247	Roots	19	0.95	11	0.55	Tonic, bone fracture	Extract
	*Saussurea lanceolata* Clarke./KNV-248	Whole plant	13	0.65	6	0.3	Skin allergies, stomach pain, typhoid	Paste, decoction
	*Taraxacum officinale* Weber./KNV-249	Whole plant	29	1.45	6	0.3	Jaundice, liver disorder, stomach pain	Poultice, juice
Balsaminaceae	*Impatiens glandulifera* Royle/KNV-292	Leaves, aerial parts	39	1.95	12	0.6	Joint pain, anxiety	Paste, extract
Betulaceae	*Betula utilis* D.Don./KNV-286	Bark	9	0.45	10	0.5	Jaundice, wound healing	Infusion, extract, poultice
Berberidaceae	*Berberis lyceum* Royle/KNV-312	Roots, leaves	41	2.05	15	0.75	Jaundice, diarrhea	Oil, juice, decoction
	*Podophyllum emodi* Royle/KNV-313	Whole plant	15	0.75	10	0.5	Cancer, jaundice, typhoid fever	Decoction, infusion
Broginaceae	*Cynoglossum lanceolatum* Forssk./KNV-319	leaves and roots	24	1.2	13	0.65	Diaphoretic, diuretic, wound healer	Oil, extract, powder
Buxaceae	*Sarcococca saligna*(D.Don) Muell/KNV-322	Leaves	19	0.95	4	0.2	Blood purifier	Extract, infusion
Caprifoliaceae	*Viburnum cotinifolium* D. Don/KNV-330	Leaves and fruits	21	1.05	5	0.25	Used for abortion and miscarriage	Powder
Chenopodiaceae	*Chenopodium album* L./KNV-331	Whole plant	17	0.85	3	0.15	Rheumatism, dysentery	Infusion, poultice, juice
Dioscoreaceae	*Dioscorea bulbifera* Decne./KNV-332	Leaves, stem, tuber	23	1.15	7	0.35	Rheumatism	Decoction, powder
Dryopteridaceae	*Dryopteris ramosa* L./KNV-344	Leaves and stem	7	0.35	8	0.4	Ulcer	Infusion, decoction
	*Dryopteris stewartii* L./KNV-345	Whole plant	11	0.55	2	0.1	Tuberculosis	Decoction
Euphorbiaceae	*Ricinus comunis* L./KNV-346	Leaves and seeds	14	0.7	7	0.35	Asthma, joint pain	Oil, poultice
Fabaceae	*Melilotus indica* (L.) All./KNV-361	Leaves and seeds	10	0.5	6	0.3	Rheumatic pains	Paste, poultice
	*Quercus ballota* (Desf.)A.DC./KNV-362	Bark	9	0.45	8	0.4	Hemorrhages, diarrhea and dysentery	Extract, decoction, infusion
	*Quercus incana* A. Camus./KNV-363	Bark and galls	18	0.9	9	0.45	Diarrhea, dysentery and throat pain	Decoction
Fumariaceae	*Fumaria officinalis* L./KNV-369	Whole plant	12	0.6	7	0.35	Skin allergies, jaundice	Decoction, powder
Gentianaceae	*Swertia petiolata* D. Don./KNV-370		12	0.6	11	0.55	Stomach pain and liver disorders	Hot infusion, juice, extract
Geraniaceae	*Geranium wallichianum* D. Don./KNV-371	Roots	6	0.3	8	0.4	Wound healing	Powder, extract
Hippocastanaceae	*Aesiculus indica* wall. Ex. Comb (Hook)/KNV-372	Seeds and fruit	10	0.5	7	0.35	Joint pain	Paste, oil
Juglandiaceae	*Juglans regia* L./KNV-373	Bark, leaves and seeds	16	0.8	3	0.15	Asthma, constipation and diarrhea	Decoction, juice
Lamiaceae	*Ajuga bracteosa* Wall. Ex Benth./KNV-384	Whole plant	18	0.9	3	0.15	Abdominal pain	Paste, powder
	*Mentha longifolia* (L.)Huds./KNV-385		14	0.7	5	0.25	Digestion	Extract
	*Plectranthus rugosus* (wall) ex Benth./KNV-386	Whole plant	19	0.95	9	0.45	Skin diseases and diarrhea	Juice
	*Prunella vulgaris* L./KNV-387	Whole plant	12	0.6	9	0.45	Heart diseases, cough and cold	Decoction
	*Salvia moorcroftiana* Wall. Ex Benth/KNV-388	leaves	3	0.15	4	0.2	Stomach pain and skin diseases	Powder, poultice
Meliaceae	*Ailanthus excelsa* Roxb.ex.Willd/KNV-229	Leaves and bark	10	0.5	6	0.3	Fever, tonic	Paste, extract
Oleaceae	*Olea ferruginea* Royle/KNV-298	Leaves	10	0.5	4	0.2	Ulcer	Decoction
Plantaginaceae	*Plantago major* L./KNV-210	Whole plant	6	0.3	4	0.2	Abdominal pain	Decoction, Powder, extract
	*Plantago ovate* Forssk./KNV-211	Seeds	13	0.65	7	0.35	Stomach disorders	Powder, paste
Polygonaceae	*Bistorta amplexicaulis* (D.Don) Greene./KNV-269	Roots	13	0.65	8	0.4	Tonic	Decoction, powder
Ranunculaceae	*Aconitum heterophyllum* Wall ex. Royle/KNV-389	Roots	7	0.35	5	0.25	Fever, cough, stomach disorders	Decoction, extract, infusion
Rosaceace	*Fragaria vesca* L./KNV-391	Leaves and fruit	5	0.25	4	0.2	Anemia	Juice, infusion, oil
	*Rubus ellipticus* Smith./KNV-392	Whole plant	5	0.25	9	0.45	Dysentery, jaundice, wound healing	Powder, paste
Rubiaceae	*Gallium boreole* L./KNV-394	Whole plant	4	0.2	7	0.35	Skin diseases	juice
Rutaceace	*Zanthoxylum armatum* DC. Proer./KNV-397	Bark, seeds and fruits	11	0.55	2	0.1	Carminative, stomachic, anthelmintic	Powder, paste, extract
Saxifragaceae	*Bergenia ciliata* (Haw.) Sternb./KNV-398	Whole plant	5	0.25	5	0.25	Asthma, stomach pain	Poultice, paste, infusion
Scrophulariaceae	*Verbascum thapsus* L./KNV-399	Leaves and flowers	8	0.4	7	0.35	Respiratory disorders	Powder, infusion
Taxaceae	*Taxus wallichiana* L./KNV-400	Bark	10	0.5	7	0.35	Cancer	Decoction
Urticaceae	*Urtica dioica* L./KNV-401	Root, aerial parts	6	0.3	6	0.3	Dandruff and baldness	Decoction

### Demographic data

Demographic data showed that there were 70% male and 30% female informants. On the basis of age, the informants were classified into four major groups, i.e., informants of 30–45 years (20%), 46–60 years (30%), 61–75 years (40%), and 76 years and above (10%). During the interview, it was observed that indigenous knowledge regarding the use of medicinal plants was more prevailing in uneducated individuals, i.e., (40%) and similar information was declining with increase in the education level of people with primary and middle level education (20%) and secondary level and graduate (10%). This might be due to the reason that illiterate people have long direct contact with medicinal plants and are well familiar to the usage of these plants. Whereas, highly educated people have little interest in traditional medicine due to their high exposure to the modern education and hence are not involved in learning and practicing ethnobotanical knowledge. Similar results were recorded by [1] in Pakistan, [39] in Ethopia, and [40] in Uganda.

### Family contribution

**Fig. 2:**
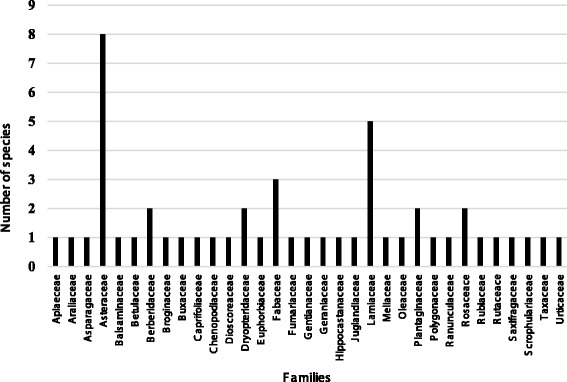
Number of species represented by each species

The most dominant family with respect to number of species used was Asteraceae (eight species) followed by Lamiaceae (five species) and Fabaceae (three species). Berberidaceae, Dryopteridaceae, Plantaginaceae, and Rosaceae were represented by two species while all other families shared one species each (Fig. 2).

The high number of species presented by these families reflects that the study area has rich biodiversity and the inhabitants have sound knowledge of the usage of these plants in locally prepared medicines. These widely distributed families in different parts of the Kel village and its surrounding areas and their extensive use might be credited to their wide range of bioactive compounds [40]. Asteraceae is largely described to have high number of bioactive compounds therefore contributing to the high use for medicinal purposes [41, 42].

### Plant part(s) used and mode of administration

**Fig. 3:**
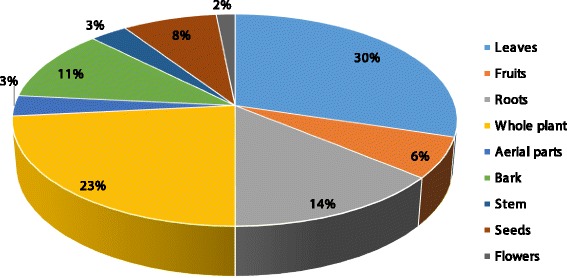
Plant parts used by traditional healers

Leaves (30%) and the whole plant (23%) were the most frequently used plant parts as shown in Fig. 3 followed by roots (14%), bark (11%), seeds (8%), fruits (6%) aerial parts and stem (3%) and flowers (2%). Leaves are extensively used in herbal medicines due to the presence of active secondary metabolites [43, 44], and many communities around the world also utilized leaves for the preparation of different herbal products [1, 2]. High proportion of leaves and roots might due to easy availability, more easily extractable phytochemicals, crude drugs, and other mixtures which have proven as valuable regarding phytotherapy [45].

**Fig. 4:**
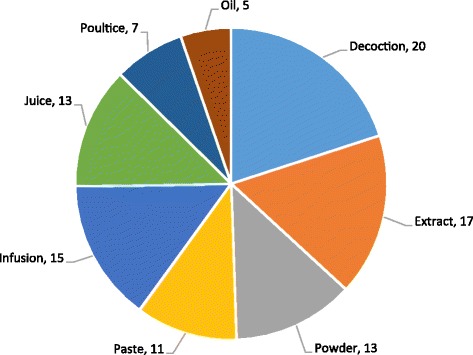
Mode of preparation of medicinal plants

Medicinal plants employed in herbal medicines were into nine classes (Fig. 4). The most common methods of preparation of herbal remedies was decoction (20%), extract (17%), infusion (15%), powder and juice (13% each), paste (11%), poultice (7%), and oil (8%). The same results were documented by [2] who reported that most plants were utilized in decoctions, followed by juice and powder. Decoctions are frequently reported as the major forms of preparation in ethnopharmacological studies because they are easy to make [2]. The powder is prepared by crushing plant parts after drying them in shade, and the paste is prepared by grinding fresh or dried plant parts with oil or water [1]. Extraction is also reported as one of the major forms of ethnobotanical practices due to the fact that during extraction, large quantities of components are released which can be used to cure ailment rapidly [46].

### Use values

A numerical method for data inquiry that measures types of uses associated with particular species and relative importance of species in a family (Vendruscolo and Mentz 2006). In our study shown in Table 2, the plant with the highest UVs was *Berberis lycium* (2.05) followed by *Impatiens glandulifera* (1.95), *Artemisia scoparia* (1.75), *Ageratum conozoides* (1.75), and *Achillea millefolium* (1.7). Other important species with respect to UVs were *Artemisia vulgaris* (1.65), *Taraxacum officinale* (1.45), *Inula grandiflora* (1.35), *Asparagus adsendens* (1.25), *Cynoglossum lanceolatum* (1.2), and *Hedera nepalensis* (1.1).

High use values of a species show its consistent use for the treatment of various diseases, and high usage reported by the number of informants shows that the plant is well recognized by the local inhabitants and is well utilized in ethomedicinal purposes [1]. The five top-ranked medicinal plants based on UVs were the mostly used plants for various ailments. Among these, *B. lyceum* was used for jaundice and diarrhea prepared in the form of paste and poultice whereas, *I. glandulifera* was commonly used for cancer and skin allergies prepared as extract. Infusion of *A. scoparia* was used to treat hepatitis, jaundice, and stomach disorders. Hot infusion of *A. conozoides* prepared with leaves or whole plant was used to cure leprosy, fevers, diarrhea, and rheumatism. Leaves of *A. millefolium* are masticated to cure toothache and juice prepared from leaves is used in earache. Tea is made from the plant as remedy to cure TB, abdominal pain, and fever. It has been observed that those plant which are used over and again are more likely to be biological active [47]. On the other hand, plant with low UVs and RFC values are not essentially insignificant. The low value may reflect the unawareness of people of that area and lack of understanding of proper use of medicinal flora [48].

### Relative frequency of citation

Relative frequency citation was calculated to determine the most common occurring medicinal plants used in various disorders as reported by the local informants. In this study, the RFC value ranges from 0.1 to 0.75 as shown in Table 2. The plant with highest RFC was *Berberis lycium* (0.75). Other significant plants with higher RFC were *Cynoglossum lanceolatum* (0.65) and *Impatiens glandulifera* and *Achillea millefolium* (0.60 each), *Ageratum conozoides*, and *Swertia petiolate* (0.55 each).

Plants with high RFC value are very famous among the local people of the area. These plants could be used in research baseline for subsequent assessment of phytochemical profiling, and in future drug discoveries [49]. Such type of plants should be exposed to further pharmacological investigations to appraise the growth of commercial yields [50]. Due to high anthropogenic pressure, such plant species should be given priority for sustainable conservation [2].

### Informant consensus factor

The Fic value ranged from 0.71 to 1.00, calculated on the base of use reports against each disease category as shown in Table 3, inferring a high consensus value among participants. The ailments were classified into 13 different categories. Disease categories urinary system, cardiac diseases, baldness, and abortion and miscarriage showed maximum (100%) consensus, since the informants approved of using only one plant species for each category. The most cited plants under these categories *Cynoglossum lanceolatum*, *Prunella vulgaris*, *Urtica dioica* and *Viburnum cotinifolium.* Other important disease categories were liver disorders and cancerous diseases with Fic values of 0.94 each, and the most cited plants for these categories were *Artemisia vulgaris* and *Taxus wallichiana*.

**Table 3:** Categories of ailments and informant consensus factor (FIC) for each category

Sample No.	Use categories	Uses under each category	Number of taxa (*N*_t_)	Number of use report (*N*_ur_)	Fic	FL	Most uses species
1	Infectious diseases	Jaundice, hepatitis, typhoid, fever, tuberculosis	7	75	0.92	95	*Berberis lyceum*
2	Disease of the ear	Earache	3	8	0.71	35	*Achillea millefolium*
3	Diseases of the circulatory system	Blood purifier, hemorrhage, anemia	3	12	0.82	55	*Fragaria vesca*
4	Diseases of the respiratory system	Asthma, cold and cough	6	74	0.93	45	*Juglans regia*
5	Diseases of the digestive system	Diarrhea, digestion, stomach disorder, ulcer, constipation, dysentery, dyspepsia	24	233	0.90	35	*Mentha longifolia*
6	Injury, poisoning and some other consequences of external cause	Skin allergies, wound healing, bone dislocation, bone fracture	11	134	0.92	60	*Fumaria officinalis*
7	Diseases of the musculoskeletal system	Rheumatism, joint pain	6	58	0.91	90	*Dioscorea bulbifera*, *Impatiens glandulifera*
8	Diseases of the urinary system	Diuretic	1	15	1.00	20	*Cynoglossum lanceolatum*
9	Cardiac diseases	Heart diseases	1	18	1.00	65	*Prunella vulgaris*
10	Liver disorders	Liver diseases	2	19	0.94	90	*Artemisia vulgaris*
11	Cancerous diseases	Cancer, tumor	3	32	0.94	35	*Taxus wallichiana*
12	Dandruff and baldness	Dandruff	1	10	1.00	30	*Urtica dioica*
13	Used for abortion and miscarriage	Used for abortion and miscarriage	1	10	1.00	60	*Viburnum cotinifolium*

The high ICF values recorded in the Kel village reflected the high dependability of native people on local flora especially for urinary, cardiac, and miscarriage diseases, whereas, low Fic values for ear diseases indicate less consistency of informer’s knowledge [51]. More often a high value of Fic is linked with few species used for one ailment group and has high use value [52], whereas a low value is linked with high number of species presenting high level of disagreement among informants to treat a specific ailment class.

Ailment classification with high Fic values prevailing in the territory may be because of the reason that native people are utilizing water sources on daily basis without having any hygienic or precautionary measurements. Disease of bronchitis, cough, flue, and fever are dominating in the mountainous areas. This is due to the reason that this area has cold and temperate type of climate, and indigenous people sustain their lives by working in forest and fields [21].

### Fidelity level

Plants were being used alone or as combined for treat different diseases. Table 3 showed the fidelity level (FL) values for species used for specific ailment as reported by the informants. Plants with higher FL value were more frequently used than those with lower Fl value. In our study, the species having the highest FL value were *Berberis lyceum* (95%) used in jaundice, hepatitis, typhoid, fever, and tuberculosis disorders, followed by *Dioscorea bulbifera*, *Impatiens glandulifera* (90%) used in rheumatism, joint pain, and *Artemisia vulgaris* (90%) used in liver disorders. *Cynoglossum lanceolatum* was the plant with the lowest FL value (25%) being used to treat urinary disorders. Plants that have higher number of FL value are considered as model plants that can be employed in further ethnopharmacological research [53].

### Direct matrix ranking

Direct matrix ranking (DMR) for tree species showed that *Olea ferruginea* had the most multipurpose use other than medicinal uses, construction, and cash income (56), firewood (28), fodder (20), fruit and food (20), and hedge and fence (12) (Table 4). Other tree species with high DMR value were *Quercus incana* and *Jaglan regia*. The lowest DMR value was recorded for *Ailanthus excelsa* (Table 5). These result indicated that native, poor people of the area are highly reliant on the tree species for construction, household furniture, fencing, and roof and wall thatching. Similar outcomes were reported by [54] in the East Zone and [55] in the North Zone of Ethopia. The multiple roles of some plants other than their medicinal significance are used in eastern medicine; nevertheless, they play an important role in the routine life of the local people [21]. Livestock raring overgrazing were common activities in the study area that is posing pressure on the flora of the region. Such activities can make the soil more compact resulting in the low seed growth and germination [56].

**Table 4:** Direct matrix ranking (DMR) of tree species with different uses other than medicinal value (total score of 10 informants) in the study area

Uses	*T. wallichiana*	*Q. ballota*	*A. excelsa*	*O. ferruginea*	*J. regia*	*A. indica*	*Q. incana*	*B. utilis*
Construction	40	49	12	56	32	55	55	48
Hedge, Fencing	18	10	20	12	07	15	15	10
Fire wood	14	55	15	28	16	12	49	26
Cash income	43	22	31	56	71	12	22	15
Fodder	9	17	15	24	20	17	34	10
Fruit, Food	08	09	04	20	34	02	08	05
Total	132	162	97	196	180	113	183	114
Rank	5th	4th	8th	1st	3rd	7th	2nd	6th

**Table 5:** Priority ranking (PR) of factors perceived as threats to plant biodiversity based on their level of destructive effects in the study area

Threats	Respondent (R1–R6)						Total	Percentage (%)	Rank
	R1	R2	R3	R4	R5	R6			
Construction	6	3	5	4	6	5	29	18.71	3rd
Timber mafia/export	5	6	4	5	7	6	33	21.29	1st
Urbanization	4	5	5	5	6	5	30	19.35	2nd
Agriculture extension	4	4	2	3	4	4	21	13.55	4th
Fuel and Fodder	3	3	2	2	4	3	17	10.97	5th

Overuse of plant resources for construction, fuel wood, and agricultural purposes made them vulnerable in the study area; thus, an urgent conservation action is needed to save the fast eroding tree flora. Necessary steps should be taken by the forest and other concerned departments, and a range land management plan should be chalk out in order to recover the local vegetation in the study area.

### PR

The priority ranking data based on the degree of threats to plants was conducted using six key informants (Table 5). In PR, informants ranked timber mafia/exports (21.2%) as a leading threat followed by urbanization (19.35%) and construction (18.71%). Other commonly prevailing threats were agriculture extension (13.55%) and fuel and fodder (10.97%). Illegal export of high-rated species was for illegitimate trading of commercial woods [57]. Several research have been carried out on the aboriginal uses of medicinal plants in Pakistan [15, 34, 58]. Hussain et al. [59] stated that the number of endangered species is increasing day by day because of overgrazing, habitat destruction, and over exploitation of medicinal plants without any conservation plan.

### Jaccard index

Ethnobotanical knowledge differs greatly between the indigenous communities because of the difference in cultural and social behaviors. Such kind of comparative analysis among communities is very useful in the sense that they can expose the significant wisdom of information among communities resulting in the findings of new drugs [60].

The results described in this our research were associated with the outcomes of 15 previous studies conducted in different surrounding areas of Pakistan, India, Nepal, and Kashmir (Table 6). The data showed that across 50 species of plants, similarity index of the data ranges from 30.77 to 1.24 whereas the variation fraction varies from 25.64 to 0.62. The maximum level of similarity was found with studies by [4] (95.65), [61] (13.04), [62] (10.57), and [7] (8.26).

**Table 6:** Jaccard index comparing the present study with previous reports at the regional, national, and global scales

Area	Study year	Number of recorded plant species	Plants with similar use	Plants with dissimilar use	Total species common in both area	Species enlisted only in aligned areas	Species enlisted only in study area	Percentage of plant with similar uses	Percentage of dissimilar uses	JI	Citation
Toli Peer A.K, Pakistan	2017	121	9	6	15	106	63	7.44	4.96	9.74	[2]
Sharda, Neelum Valley	2012	39	12	10	22	17	28	30.77	25.64	95.65	[4]
Abbottabad, Northern Pakistan	2014	120	2	4	6	114	44	1.67	3.33	3.95	[1]
Senhsa, District Kotli A.K, Pakistan	2012	112	6	7	13	99	37	5.36	6.25	10.57	[62]
Hangu North KPK Pakistan,	2014	67	1	1	2	65	48	1.49	1.49	1.80	[68]
Kashmir Himalayas, Pakistan	2012	71	3	4	7	64	43	4.23	5.63	7.00	[36]
Central Karakoram national Park, Gilgit-Baltistan, Pakistan	2011	47	1	1	2	45	48	2.13	2.13	2.20	[70]
Naran Valley, Pakistan	2013	101	5	5	10	91	40	4.95	4.95	8.26	[7]
Garhwal Himalaya, India	2013	152	2	6	8	144	42	1.32	3.95	4.49	[71]
District Bhimber A.K, Pakistan	2013	58	1	1	2	56	48	1.72	1.72	1.96	[22]
Leepa Valley, Muzaffarabad (AJK), Pakistan	2012	36	3	1	4	32	46	8.33	2.78	5.41	[21]
Makawanpur District, Central Nepal	2014	161	2	1	3	158	47	1.24	0.62	1.49	[63]
Terai forest, Western Nepal	2012	66	2	1	3	63	47	3.03	1.52	2.80	[64]
Rasuwa District, Central Nepal	2010	60	3	1	4	56	46	5.00	1.67	4.08	[65]
Jakholi block, Rudraprayag, India	2017	78	8	4	12	66	38	10.26	5.13	13.04	[61]

The highest degree of similarity index was found in the study of [4], which revealed the same ethnic values and the same type of vegetation and geography of both areas. Furthermore, cultural exchange could have been occurred in the past between the indigenous communities resulting in the similarity in the ethnobotanical outcomes of both areas [2]. *Berginia ciliata*, *Juglan regia*, *Xanthoxylum armatum*, *Taxus wallichiana*, *and Ageratum conozoides* from various districts of Central and Western Nepal [63–65] were reported with similar uses. This reflected the similar climatic conditions of the adjacent areas [66] and also high adaptability of these plants to grow in different ecological zones at various elevations [67].

The minimum JI value was recorded for the work conducted by [63] (1.49) in Makawanpur District, Central Nepal [68] and in Hangu, North KPK, Pakistan, and [22] (1.96) in District Bhimber A.K, Pakistan. This difference in ethnobotanical knowledge might be because of the presence of some ecological barrier resulted in geographic isolation of species [1], and the diversities in vegetation and habitats [2]. Beside habitat isolation and difference in vegetation type, researchers have also found that ethnobotanical knowledge vary with age, sex, education level, and origin of the informant [69]. It can be concluded from these studies that geographical isolation among communities has great impact on change in vegetation type and transformation of cultural knowledge, and this might be a cause of the loss of ethnobotanical knowledge.

### Pearson correlation analysis

**Fig. 5:**
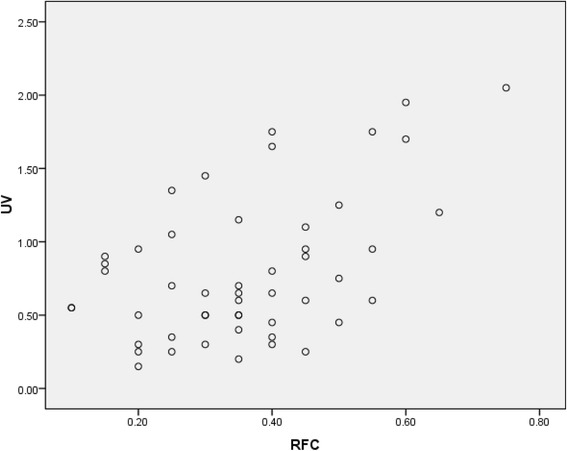
Association between use value and relative frequency of citation

Pearson analysis method was used to measure the strength and direction between UV and RFC variables. Both UV and RFC variables (0.500) had positive relationship of species uses by the number of informants (Table 7, Fig. 5). The coefficient of determination value (*R* ^2^ = 0.25) showed 25% of the difference in use value of species with respect to their citations. This indicated that this work has significant contribution in the documentation of ethnobotanical information on traditional plant uses [22, 57].

**Table 7:** Correlation between use value and relative frequency of citation

	Correlations	UV	RFC
UV	Pearson correlation	1	.500^a^
	Significance (two-tailed)		.000
	*N*	50	50
RFC	Pearson correlation	.500^a^	1
	Significance (two-tailed)	.000	
	*N*	50	50

^a^Correlation is significant at the 0.01 level (two-tailed)

*R* ^2^ = 0.25

### Novelty and future impact

The present study was compared with the previous studies conducted in different parts of the Himalayan territory and other areas as shown in Table 6, to find the novelty index. Medicinal uses of plant species reported in neighboring areas showed more resemblance compared to those documented in other areas. The data collected from the study area reveal considerable difference in plant parts used, mode of herbal preparation, and its utilization as reported from other regions.

Some of the newly documented medicinal uses and relevant plant species include *Hedera nepalensis* (ulcer), *Inula grandiflora* (liver pain), *Jurinea dolomiaea* (bone fracture), *Saussurea lanceolata* (typhoid), *Impatiens glandulifera* (joint pain), *Betula utilis* (Jaundice), *Podophyllum emodi* (cancer), *Dryopteris ramosa* (ulcer), *Dryopteris stewartii* (tuberculosis), *Quercus ballota* (dysentery), *Swertia petiolata* (liver pain), *Fumaria officinalis* (skin allergies), *Plectranthus rugosus* (skin allergies and diarrhea), *Prunella vulgaris* (heart diseases), *Ailanthus excelsa* (fever), *Bistorta amplexicaulis* (tonic), *Rubus ellipticus* (wound healing), and *Gallium boreole* (skin problems). The plant species with new medicinal uses and high RPL value could be studied further to screen bioactive compounds and their pharmacological activities to introduce novel drugs.

## Conclusion

This study demonstrates that many plant species play an important role in local healing practices and that knowledge of traditional medicine is still utilized and plays a significant role on Batan Island. The documentation of this rich traditional ethnomedicinal knowledge has furnished us with novel information that will provide recognition of this undocumented knowledge. The natives of the region are very much reliant on the local medicinal plant to encounter health needs, fuel wood, and fodder. The data show that practicing ethnobotanical knowledge significantly differ within the region and around the globe in terms of preparation of herbal drugs, thus providing fresh ethnomedicinal knowledge. Plants with high use report such as *Berbaris lyceum*, *Impatiens glandulifera*, *Artemisia scoparia*, *Ageratum conozoides*, *Achillea millefolium, Artemisia vulgaris*, *Taraxacum officinale*, *Inula grandiflora*, *Asparagus adsendens*, *Cynoglossum lanceolatum*, and *Hedera nepalensis* have great potential to be used in further ethnopharmacological studies. Most of the plants in the area are endemic facing several threats such as over exploitation, deforestation, and overgrazing need immediate conservation. It is also suggested that biotechnological methods, e.g., tissue culture, micropropagation, synthetic seed technology, and molecular marker-based studies, should be to improve healthcare for a range of ailments.

This study will help us to link ethnobotanical and chemical knowledge to understand the use of medicinal plants by traditional communities. The information obtained from this study will encourage native communities in trading off locally prepared herbal products. As a result of expanding interest, new income-generating opportunities will be available for poor rural household. Moreover, sustainable uses of plant resources will promote biological and cultural diversity which in return will promotion of local biocultural diversity through ecotourism initiatives.

## Abbreviations

DMR: Data matrix ranking
Fic: Informant consensus factor
FL: Fidelity level
IPNI: International Plant Name Index
JI: Jaccard index
PR: Priority ranking
RFC: Relative frequency of citation
UV: use value
